# Long-term performance of ceramic in/-onlays vs. cast gold partial crowns – a retrospective clinical study

**DOI:** 10.1007/s00784-024-05682-7

**Published:** 2024-05-03

**Authors:** Ralf Krug, Lea Droste, Carolina Schreiber, Elisabeth Reichardt, Gabriel Krastl, Britta Hahn, Sebastian Soliman

**Affiliations:** 1 Department of Conservative Dentistry and Periodontology and Center of Dental Traumatology, University Hospital of Würzburg, Würzburg, Germany; 2 Private Practice, Düsseldorf, Germany; 3 Private Practice, Brunswick, Germany; 4 Department of Dentistry and Oral Health, Aarhus University, Aarhus, Denmark

**Keywords:** Ceramic, Cast gold, Partial crown, Inlay, Onlay, Success, Survival rate, USPHS criteria

## Abstract

**Objectives:**

To assess the long-term clinical performance of ceramic in-/onlays (CIOs) and cast gold partial crowns (CGPCs) in posterior teeth in terms of success, survival, complications (biological, technical) and quality.

**Material and methods:**

In a retrospective study, a total of 325 patients were recorded after up to 24.8 years (mean 13.9 ± 3.8 years) having (pre-)molars restored with CIO (Empress I, Ivoclar Vivadent, *n* = 161) and CGPC (Degunorm, DeguDent, *n* = 164) by supervised undergraduate students. A total of 296 restorations were assessed clinically and radiologically in healthy and endodontically treated teeth using modified United States Public Health Service (USPHS) criteria. Cumulative success and survival rates of the restorations were calculated using Kaplan–Meier estimates. Biological and technical complications were recorded. Status of oral health comprising caries risk and localized periodontitis were assessed.

**Results:**

The cumulative success rates of CIOs were 92.1% and of CGPCs 84.2% after mean service times of 14.5 years. The annual failure rates of total service times were 0.5% in teeth restored with CIO (*n* = 155) and 0.7% in teeth restored with CGPC (*n* = 163). The cumulative survival rates of CIOs were 93.9% after a mean service time of 15.2 years and decreased to 91.7% after 23.5 years. The cumulative survival rates of CGPCs were 92.6% after a mean service time of 14.9 years and 91.8% after 23.5 years. Complications in CIOs (*n* = 149) were ceramic fracture (6.7%), secondary caries (4.7%), endodontic complication (2.7%) and tooth fracture (1.3%) compared to CGPCs (*n* = 147) with endodontic complication (8.8%), secondary caries (4.8%) and decementation (2.0%). Endodontically treated teeth restored with CIO or CGPC revealed significantly less often success compared with corresponding vital teeth (*p* = .02). CIOs and CGPCs revealed clinically and radiographically good and excellent qualities with 71.8% (107/149) and 68% (100/147) without any significant differences regarding type of restoration.

**Conclusions:**

Both CIOs and CGPCs achieved high survival rates up to 24.8 years when performed by supervised undergraduate students. The longevity of the restorations may benefit from the intraoral repair of accessible defects and, in case of pulp infection or necrosis, an adequate endodontic management.

**Clinical relevance:**

CIOs and CGPCs made by supervised undergraduate students are proper restoration types in posterior teeth in the long-term. An adequate preparation design, meticulous care in the inserting technique and constant biofilm removal due to proper oral hygiene combined with professional maintenance care are substantial. The clinical long-term performance was mostly limited by ceramic fractures in CIOs and endodontic complications in CGPCs.

## Introduction

Partial crowns, in- and onlays are common types of indirect restorations in posterior teeth. They are usually manufactured from gold alloys or ceramics, nowadays sometimes from composite or polymer-infiltrated ceramics. They allow a functional reconstruction of large defects while preserving more sound hard tissue compared to full crown coverage [1]. Endodontically treated premolars and molars may benefit from this approach since partial crowns are believed to reinforce such teeth by minimizing diverging forces impacting the cups which might lead to tooth fracture [2–4]. Consequently, lower mean fracture rate values were reported in endodontically treated teeth restored with indirect restorations compared to those with direct restorations [5]. Previous studies revealed a lower annual failure rate for indirect restorations in posterior teeth compared to direct restorations [6, 7]. Recent investigations showed rather similar annual failure rates for both restoration types (direct: 1.1%; indirect: 1.6%) [8, 9]. However, a recent meta-analysis assessing the clinical performance of composite and ceramic restorations showed that the failure risk increased with defect size. Further, one common complication was the fracture of the ceramic, whereas resin composite materials mainly failed due to secondary caries [10]. If adhesive techniques with cusp-replacement are used, the performance of direct postendodontic restorations seems to be almost equivalent to that of indirect techniques [11]. From a clinical perspective, indirect restorations can be considered to be superior to direct fillings in terms of marginal adaption, polishing, and design of the proximal contact area [12].

Esthetic requirements play a crucial role in selecting the most suitable type of restoration. Tooth-colored materials allow a wide range of reconstructions complying with highest esthetic demands. Further, the preparation design can be extended on buccal tooth cusps and surfaces. Nowadays, when restoring teeth with cast gold partial crowns (CGPC), the extension of the preparation should be limited to esthetically insignificant tooth surfaces resulting in a more challenging tooth preparation.

Gold alloys are characterized by two substantial longevity-related dental material properties: the plastic deformation of its metallic microstructure and the effect of increased hardness after deformation. In a restoration’s lifetime occlusal forces and recurrent occlusal wear deform the metallic surface. Thus, a micro-mechanical adaptation might rather resist the effects of abrasion and attrition in the long-term. That is why the fracture of an alloy can be almost excluded as a complication of clinical relevance. Ceramics have different properties and limitations, irrespective of their various fabrication methods: the proneness for cohesive and adhesive fractures, a high brittleness, a high bending strength, a low toughness, and specific handling requirements regarding the design of prepared cavity or adhesive luting method [13, 14]. Several studies investigated the beneficial effects of adequate ceramic thickness with a minimum of approximately 1.5 mm, cusp coverage preferred in endodontically treated teeth, adequate remaining cusp wall thickness with at least 2.0 mm, and proper ceramic surface processing in order to reduce failures such as crack formation [15–19]. However, there is much evidence in literature, that fracture poses the most common failure of ceramics [20–22]. Other adverse events are secondary caries, retention loss, and delayed endodontic treatment for both restoration types [23–32]. Nevertheless, the crucial advantages of partial crowns made of gold alloys or ceramics are the high amount of preserved sound dental hard tissue and the excellent biocompatibility [33].

Many different patient- and operator-related factors affect the longevity of an indirect restoration. Restorative aspects are an extensive loss of dental hard tissue in posterior teeth, a cost–benefit-analysis balancing other treatment options, and the feasibility of the adhesive cementation. Several clinical studies revealed favourable rates of survival in vital teeth restored with CGPCs compared to similar rates in those with ceramic partial crowns (CPCs) [23, 30, 31], in endodontically treated teeth as well [34]. Survival estimates were calculated after 13 years with 72% in CGPCs from 42 patients and after 7 years with 81% in CPCs from 22 patients [30]. One prospective split-mouth study showed a cumulative survival rate after 5.5 years with 93.3% in CGPC and 88.8% in CPCs from 29 patients [31]. Interestingly, the pooled estimated 10-year survival rate of ceramic inlays, onlays and overlays (*n* = 2154 restorations) was calculated with 91% regardless of the material (glass ceramic or feldspathic porcelain), study design or setting [20]. Whereas the range of Kaplan–Meier-survival estimates in teeth with CGPC revealed 72 to 98.9% [24, 25, 30, 31, 35], the survival for ceramic restorations after 4 to 8 years was 81 to 92% [29–31, 36–38] and up to 18 years with 75.9 to 92.4% [26–28, 39, 40]. A drawback of clinical studies calculating cumulative survival rates remain short-term observation data and small sample sizes of patients/restorations in a selected pool.

This retrospective study aimed to evaluate the long-term performance of CGPCs and ceramic in-/onlays (CIOs) with the primary outcomes of calculating the cumulative success and survival rates. Secondary outcomes were quality and complications (biological, technical). The study collective was a patient pool treated within one consistent university teaching from two decades.

## Material and methods

Three-hundred-twenty-five study participants were recruited from a pool of 1651 patients, who received CGPCs (Degulor C) and CIOs (leucite reinforced glass ceramic) between the years 1994 and 2009 at the Department of Conservative Dentistry and Periodontology, University Hospital of Würzburg, Germany. All restorations were performed by supervised fifth-year students in vital and endodontically treated teeth. Ethical approval (no. 184/15) was obtained from the local ethics committee for the clinical and radiographic evaluation and all participants provided written informed consent. There was a total of 1126 patients meeting the inclusion criteria (Table 1). A patient’s restoration was excluded in case of less than three years of service time in order to exclude any technical short-term failures. A total of 600 patients was successfully contacted by phone on two occasions or once by mail. Three hundred and twenty-five patients were willing to make an appointment for the follow-up examination (Table 2), performed by two dentists (L.D.,C.S.) achieving a consensus. The clinical examination started with two calibration-set-ups of all variables for the first ten restorations of each type (CGPC/CIO) in coordination with a certified university’s principal investigator (R.K.) with ten years of experience in restorative dentistry. The presence or absence of clinical signs and symptoms were assessed using pain, discomfort, sensitivity to percussion and pulp vitality, pocket probing depth (PPD) and clinical attachment level (CAL). Further, tooth mobility index by Lindhe & Nyman (1977), sulcus bleeding index (SBI) compassing six measuring points per tooth by Mühlemann & Son (1971) and the modified Plaque-Index by Turesky (1970) were assessed. Caries risk assessment was performed according to Hotz et al. (2005) and categorised into three degrees. Digital radiographs (VistaScan, Duerr Dental SE, Bietigheim-Bissingen, Germany) were made to evaluate the presence of periapical lesion, secondary caries and quality of the restoration.

**Table 1:** Inclusion criteria for the retrospective study within a university teaching

patient’s age	18 to 85 years
restoration	CGPC (cast gold partial crown) or CIO (ceramic in-/onlay with at least one cusp replaced)
	inserted between 1994 and 2009
operator	a fifth-year undergraduate student

**Table 2:** Distribution of the selected patient pool with type of tooth/restoration and drop-outs

	*n*	%
recall rate	325/1126	28.90
patients with informed consent	325	100.00
cast gold partial crown (CGPC)	164	50.46
molars	147	49.54
premolars	17	
ceramic in-/onlay with at least one cusp replaced (CIO)	161	
molars	115	19.88
premolars	46	
CIO restored with 3-surfaces	32	
4-surfaces	76	47.20
5-surfaces	53	32.92
drop-out due to tooth extraction	17	5.23
restoration not in situ	12	3.70
patients with complete clinical and radiological examination of the restored tooth in situ	296	91.07

The CGPCs were manufactured by using high-gold alloy (Degulor C, Degudent, Dentsply Sirona, Bensheim, Germany) and inserted with glass ionomer cement (Ketac™ Cem, 3 M, Neuss, Germany). The CIOs were made of leucite reinforced glass ceramic (Empress I, Ivoclar Vivadent AG, Schaan, Liechtenstein) and inserted with an adhesive resin cement (to 54.2% with Bifix QM, VOCO GmbH, Cuxhaven, Germany; to 38.3% with Compolute™, 3 M, or Variolink, Ivoclar Vivadent AG) or by using acid-etch-technique with a flowable composite (Tetric EvoFlow, Ivoclar Vivadent AG). During inserting the CIOs rubber dam was used obtaining moisture-free environment. The placement of partial crowns was usually performed by fifth-year students supervised by university’s dentists. A total of 13.5% (44/325) of the student’s restorations were inserted by the dentist, predominantly in challenging cases.

In case of CGPC the cusps from maxillary teeth were covered up to the buccal ridge line, cusps from mandibular teeth were minimally extended on the external surface. In the case of ceramics at least one cusp-replacement was needed to include it in this study assessing different types of CIO with three up to five restored tooth surfaces. At follow-up patients had a mean age of 59.2 ± 10.8 years. The mean observation times were 13.8 ± 4.1 years for CGPCs (*n* = 164) and 14.0 ± 3.5 years for CIOs (*n* = 161, 3 missings).

Modified United-States-Public-Health-Service (USPHS)-criteria (in total 13) were used for evaluation of restorations’ quality with a range of 1 to 5 scores (Hickel et al. 2007) (Table 3). Two categories were defined subdividing various quality levels (Table 4). All restorations of a patient were systematically listed. CGPCs and CIOs with earliest date of placement were selected obtaining one restoration for each patient. Complications with the time point of intervention, type and diagnosis during and after placement of the selected restoration were collected from the patient records or the attended dentist.

**Table 3:** Clinical and radiological examination using modified USPHS-criteria (Hickel et al. 2007)

criteria	method of examination	score	
esthetic characteristics		CGPC	CIO
surface quality / polishing	visual-tactil	1–5	1–5
discoloration of surface / margin	Visual	1–5	1–5
colour stability	Visual		1–5
anatomic shape	Visual	1–5	1–5
functional characteristics		CGPC	CIO
surface defects / retention	visual-tactil	1–5	1–5
quality of margins	tactil	1–5	1–5
quality of promixal contact	visual and mechanically using dental floss	1–5	1–5
radiological examination	assessment of periapical radiograph	1–5	1–5
patient’s satisfaction	patient interview	1–5	1–5
biological characteristics		CGPC	CIO
sensitivity/ endodontic status	cold test / radiological evaluation	1–5	1–5
caries, erosion	visual-tactil	1–5	1–5
periodontal probing	visual/mechanically using periodontal probe instrument	1–5	1–5
muco-gingival status	Visual	1–5	1–5
	total	50	55

**Table 4:** Categories (A,B) assessing quality of CIO and CGPC

quality	single characteristic	sum of scores	type of restoration
category A			
high	≤ 3	≤ 30	CGPC
		≤ 33	CIO
poor	> 3	> 30	CGPC
		> 33	CIO
category B			
excellent	≤ 3	≤ 13	CGPC
		≤ 14	CIO
good	≤ 3	14—20	CGPC
		15—22	CIO
acceptable	≤ 3	21—30	CGPC
		23—33	CIO
deficient	> 3	> 30	CGPC
		> 33	CIO
insufficient	> 4	> 30	CGPC
		> 33	CIO

The dichotomized variable “oral health” was defined based on the findings of evident gingivitis (SBI values ≥ 10%), high plaque accumulation (PI > 3), and increased caries risk (≥ 2) for diagnosing poor oral health. Localized periodontitis was detected in the region of both the restored tooth and the adjacent teeth if signs of increased tooth mobility (≥ 2) and/or increased PPD (≥ 5 mm) were present compared critically with radiological findings of pathologic bone loss. Additionally, data were categorised in terms of the tooth type (premolar or molar) and a dichotomized variable regarding vital or non-vital tooth (with endodontic complication).

A total of *n* = 325 patients were used for descriptive statistics and the calculation of Kaplan–Meier success and survival rates based on treatment outcomes (Table 5). Esthetic, functional, and biological characteristics were assessed in restored teeth of examined patients at follow-up (*n* = 296). Statistical analyses were performed with SPSS software (Vers. 28.0.1.1, IBM Corp., Armonk, USA). Chi-square-tests for independence with Yates Continuity Correction were conducted on 2 × 2 contingency tables showing significant differences of non-metric scaled data. Effect sizes were expressed as Phi (φ) or Cramer’s *V*. Binary logistic regression analyses were conducted to test if there are specific predictors for success or non-success. Cox & Snell R^2^ and Nagelkerkes R^2^ indicated the model’s variance of dependent variables. Kaplan–Meier analysis were represented graphically to point out complications within the selected observation period. The use of the log-rank test allowed comparing success and survival rates of CGPCs and CIOs as unpaired samples. The null hypothesis, that posterior teeth restored with either CIO or CGPC do not differ in terms of success, survival, failure or quality of the restoration in the long-term, was tested. The level of statistical significance was set at α = 0.05.

**Table 5:** Definition of treatment outcomes

success	no complications, restored tooth in function
survival	complications required intervention/repair, but restored tooth in function
failure	restoration lost, tooth restorable or restored with new restoration, tooth lost or extracted

## Results

Excellent and good qualities were assessed up to 68% (100/147) in CGPCs and 71.8% (107/149) in CIOs. There were no significant differences between the quality of CGPC and that of CIO for category A (χ^2^(1, *n* = 296) = 1.51, *p* = 0.22, *φ* = -0.07) and category B (χ^2^(4, *n* = 296) = 6.6, *p* = 0.16, Cramer’s* V* = 0.15).

The restorations of the recalled patients revealed similar success rates with 75.6% (124/164) in case of CGPC (range of follow-up: 3.2 to 24.3 years) and 78.3% (126/161) in case of CIO (3.3 to 24.8 years) (Table 6). There was no significant correlation between the prevalences of success and survival compared with the types of restoration, χ^2^ (3, *n* = 325) = 7.51, *p* = 0.06. There was a significant correlation between failure and the type of restoration, *χ*^2^ (1, *n* = 46) = 4.62, *p* = 0.03, φ = 0.36. Posterior teeth with CGPC were rather extracted than those with CIO, whereas teeth with CIO rather achieved a new restoration than those with CGPC. Endodontic failure was the most common reason for failure in teeth restored with CGPC (29.4%, 5/17). Except for vertical bone loss, other reasons were not recorded. No reasons were identified for extraction of all teeth with CIO restored (*n* = 5). Secondary caries was the main reason for re-restoring teeth with a new CGPC (5/10). Correspondingly, there were 14 teeth with CIO which received a new restoration. Three cases (3/14) were affected by secondary caries and another three (3/14) by ceramic fracture. Further reasons in remaining cases (8/14) were not recorded.

**Table 6:** Distribution of treatment outcomes in the recalled patients (*n* = 325)

type of restoration	treatment outcome							
	success		survival		failure		total	
	*n*	%	*n*	%	*n*	%	n	%
CGPC	124	38.2	13	4.0	27	8.3	164	50.5
CIO	126	38.8	16	4.9	19	5.8	161	49.5
total	250	77.0	29	8.9	46	14.1	325	100

There was no significant correlation between type of tooth (molar, premolar) and treatment outcomes, *χ*^2^ (2, *n* = 325) = 1.69, *p* = 0.43. Within the data of both CGPC and CIO no significant correlations were between the type of tooth (molar, premolar) and treatment outcomes, CGPC: *χ*^2^ (2, *n* = 164) = 1.63, *p* = 0.44. CIO: *χ*^*2*^ (2, *n* = 161) = 2.01 Pearson, *p* = 0.37

The category failure was detected after a mean service time of 11.3 ± 4.4 years (*n* = 42, 4 missings). Kaplan–Meier estimates were compiled comparing the periods of success or survival between CGPC and CIO. In terms of a 20-year service time 84% (137/163) of CGPCs and 90.3% (140/155) of CIOs had to be censored due to shorter times of follow-up estimating success. Estimating survival, 92.6% (151/163) of CGPCs and 92.9% (144/155) of CIOs had to be censored due to shorter times of follow-up, respectively.

The null hypothesis was not rejected as the log-rank-test revealed no significant difference regarding the category success between CGPCs and CIOs, *χ*^*2*^ (1, *n* = 311 (CIO: 149, 6 missings; CGPC: 162, 1 missing)) = 2.85, *p* = 0.091.

**Fig.1:**
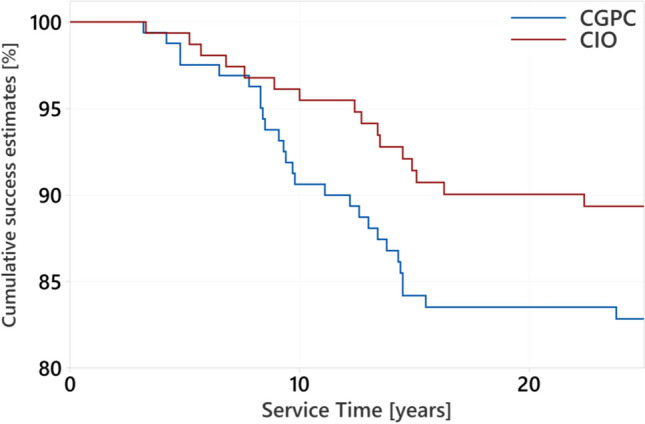
Kaplan–Meier success estimates in teeth restored with CGPC (*n* = 163) or CIO (*n* = 155)

The cumulative success rates of CIOs were 92.1% after a mean service time of 14.5 years and decreased to 89.3% after 22.4 years. The cumulative success rates of CGPCs were 84.2% after a mean service time of 14.5 years and 82.9% after 23.8 years, respectively (Fig. 1). The annual failure rates of total service times were 0.5% in teeth restored with CIO and 0.7% in teeth restored with CGPC.

**Fig.2:**
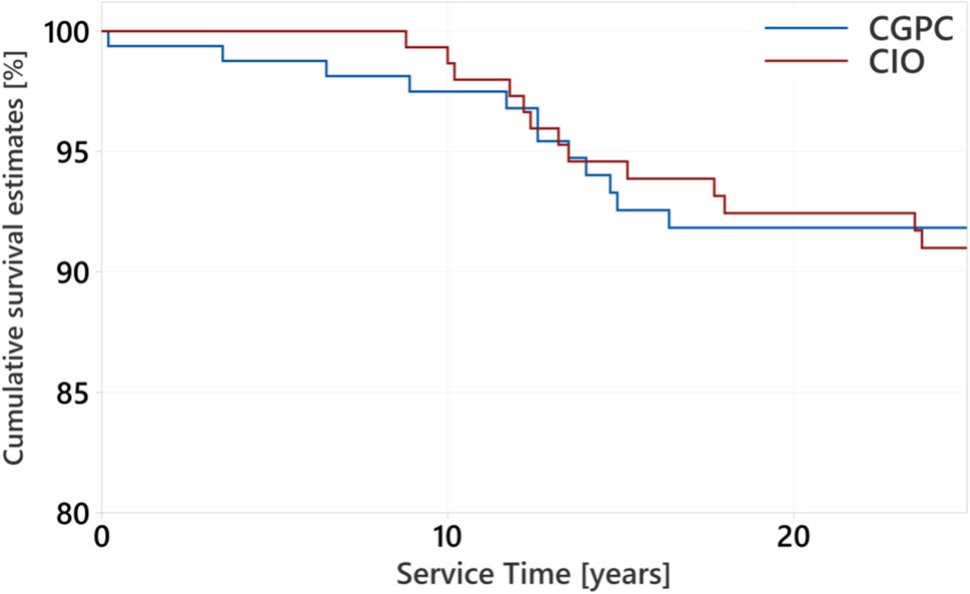
Kaplan–Meier survival estimates in teeth restored with CGPC (*n* = 163) or CIO (*n* = 155)

The cumulative survival rates of CIOs were 93.9% after a mean service time of 15.2 years and decreased to 91.7% after 23.5 years. The cumulative survival rates of CGPCs were 92.6% after a mean service time of 14.9 years and 91.8% after 23.5 years, respectively (Fig. 2).

Of all teeth 84.5 ± 0.1% were free of complications. Endodontic complications were more often found in CGPCs (8.8%) than in CIOs (2.7%). Secondary caries was approximately to 4.7% in both types of restoration (Table 7). Survival of restorations was not associated with endodontic complication, rather with secondary caries and ceramic fracture (Table 8). All fractures of CIOs were detected in vital teeth (10/137). Complications were almost equally distributed to 22.9% (60/262) in molars and to 23.8% (15/63) in premolars. There were defects in need of repair up to 20.8% (31/149) in CIOs and 25.9% (38/147) in CGPCs.

**Table 7:** Distribution of detected complications in teeth (*n* = 296) restored with CGPC and CIO

	CGPC		CIO	
	n	%	N	%
no complication	124	84.35	126	84.56
complication				
endodontic	13	8.84	4	2.68
secondary caries	7	4.76	7	4.70
decementation/debonding	3	2.04		
fracture of restoration			10	6.71
fracture of tooth			2	1.34
total	147		149

**Table 8:** Distribution of different complications in terms of survival with repair and failure in teeth (46/296) restored with CGPC and CIO

treatment outcome	type of complication											
	endodontic complication		secondary caries		tooth fracture		ceramic fracture		decementation		total	
	*n*	%	*n*	%	*n*	%	*n*	%	*n*	%	*n*	%
survival with repair	12	26.1	5	10.9	2	4.3	7	15.2	3	6.5	29	63
failure	5	10.9	9	19.5	0	0	3	6.5	0	0	17	37.0
total	17	37.0	14	30.4	2	4.4	10	21.7	3	6.5	46	100

A logistic regression was conducted in order to test a model predicting failure in teeth restored with CGPCs or CIOs depending on following factors: complication, secondary caries, endodontic complication, patient’s age, age of restoration, and gender. This model was significant with all six predictors, χ^2^ (6, *n* = 296) = 77.84,* p* < 0.01. It ranged between 23.1% (Cox & Snell R^2^) and 65% (Nagelkerkes R^2^). It met the correct category with a rate of 96.3%. In case of secondary caries as the one significant predictor of this model the probability of predicting “no success” increased with an odds ratio of 6.6 (Table 9).

**Table 9:** Evaluation of variables for predicting the outcome failure in teeth restored with CGPC or CIO using binary logistic regression (CI = confidence interval)

Variable	*B*	*SE*	Wald	*df*	*p*	Odds ratio	95% CI	
							lower	upper
complication	19.92	2520.93	0	1	0.99	4.50E + 08	0	
secondary caries	1.89	0.89	4.47	1	0.03	6.60	1.1	37.9
endodontic complication	0.22	0.88	0.06	1	0.81	1.24	0.2	6.9
age of restoration	-0.10	0.09	1.19	1	0.28	0.91	0.8	1.1
patient’s age	0.02	0.04	0.34	1	0.56	1.02	0.9	1.1
gender	-0.22	0.68	0.10	1	0.75	0.80	0.2	3.1

Endodontically treated teeth restored with CGPC or CIO showed low rates of failures irrespective of treatment pre- or postoperatively (Table 10). There was a significant difference between the outcome “success or survival” in contrast to failure and performed endodontic therapy in restored teeth, χ^2^ (2, *n* = 296) = 7.36, *p* = 0.02, Cramer’s *V* = 0.16.

**Table 10:** Distribution of restored teeth with various endodontic status in terms of treatment outcome (RCT = root canal treatment)

endodontic status	treatment outcome					
	success or survival		failure		total	
	*n*	%	*n*	%	*n*	%
vital tooth, no need for RCT	237	80.1	12	4.1	249	84.2
RCT before placement of restoration	37	12.5	3	1	30	13.5
RCT after placement of restoration	5	1.7	2	0.6	7	2.3
total	279	94.4	17	5.6	296	100

Regarding the evaluation of biological complications affecting treatment outcome the variable „oral health “ was assessed in examined teeth (*n* = 295, 1 missing). There was a homogenous distribution of the patients in terms of „oral health “ with the different treatment outcome for both restoration types (CGPC and CIO) (Table 11). The absence of secondary caries in restored teeth was detected in 95.1% (235/247) of the patients with „good oral health “ and in 95.8% (46/48) of the patients with „poor oral health “. There was no significant correlation in patients with poor oral health between the type of restoration (CGPC or CIO) and the presence of secondary caries, *χ*^*2*^ (1, *n* = 48) = 0.96, *p* = 0.33.

**Table 11:** Distribution of status of the variable „status of oral health “ in terms of treatment outcome in teeth restored with CGPC and CIO (*n* = 295, 1 missing)

status of oral health	type of restoration	treatment outcome							
		success		survival		failure		total	
		*n*	%	*n*	%	*n*	%	*n*	%
good oral health	CGPC	100	33.9	10	3.4	9	3.1	119	40.3
	CIO	108	36.6	14	4.7	6	2.0	128	43.4
poor oral health	CGPC	24	8.1	3	1	1	0.3	28	9.5
	CIO	17	5.8	2	0.7	1	0.3	20	6.8
total		249	84.4	29	9.8	17	5.7	295	100

Additionally, the impact of localized periodontitis in the region of the restored tooth on complication rate and the treatment outcome were evaluated. There was no significant correlation between the presence of localized periodontitis and the occurrence of complication in restored teeth, χ^2^ (1, *n* = 295) = 0.13, *p* = 0.72. Further, there was no significant correlation between the presence of localized periodontitis and failure of restoration, χ^2^ (1, *n* = 295) = 3.19, *p* = 0.07.

## Discussion

The present study revealed high cumulative success rates with 92.1% and 84.2% after mean service times of 14.5 years in posterior teeth restored with CIO (leucite reinforced glass ceramic) or CGPC (high gold alloy) performed by supervised undergraduate students. There were similiar annual failure rates of total service times with 0.5% and 0.7%, respectively. The cumulative survival rates were 81.3% in CIOs after 23.7 years and 76.1% in CGPCs after 23.8 years. Of all examined teeth, 84.5% stayed free of complication. There were excellent and good qualities with a rate of 71.8% in teeth restored with CIO and 68% in teeth restored with CGPC.

The recall rate was 28.9% (325/1126), which is a common drawback of retrospective clinical studies [41–44]. A low recall rate may limit the results due to a high drop-out rate as a common effect in long-term observational studies. Various drop-out rates in long-term studies were reported with 12.3% after a mean observation period of 18.7 years [24] assessing gold restorations, with 9.5% after 15 years [28] in a prospective study assessing ceramic restorations or up to 40% after eleven [45] and twelve years [26] in retrospective studies. In the present study, randomly selected and non-specific patients were examined, in contrast to other studies excluding patients with poor oral hygiene and bruxism [26, 38, 46, 47], with high caries risk [48], or with endodontically treated teeth [46]. Within the limitations of the high drop-out rate and the retrospective study design, the included patient pool of this study seems to represent general practice patients with various risk factors. Each patient was invited to be part of a systematic follow-up examination with professional tooth cleaning service or, if necessary, a systematic treatment of periodontitis. In the present study, 83.7% of the recalled patients showed a status of good oral health. Secondary caries was with a rate of 4.7% very low for both types of restoration. It can be assumed that there was a beneficial effect of good oral hygiene supporting the effect of caries prevention.

Selected study criteria aimed to exclude technical short-term failures, which can be caused by procedural errors from the operator or the dental technician. Failures do also occur due to inadequate properties of the dental material. In the present clinical study, the adhesive cementation was always performed using rubberdam in order to minimize the risk of debonding in CIOs. Other key aspects of the present study were a well-established and standardized university’s training concept since nearly two decades, the systematic support of supervising dentists, and the entire dental technical work supported by two experienced dental technicians (K.H., J.M.) from an in-house dental laboratory. Thus, all indirect restorations were fabricated and inserted with the claim for a high level of accuracy resulting in a clinical performance as good as possible. The present study showed excellent and good qualities with 68% (in CGPCs) and 71.8% (in CIOs) for both types of restoration. There are no differences regarding the clinical performance or the survival of ceramic restorations in data pools from university teaching compared with data pools from private practice [49].

In literature, there are numerous short-term and a few long-term data with high survival rates for teeth restored with ceramic partial crowns, mostly up to ten years. It was also reported that includable information on the survival of ceramic on- and overlays performing up to 15 years are barely available [49].

Numerous retrospective studies revealed survival data of partial crown coverage in posterior teeth with a high number of recalled patients [23, 24, 27, 29, 30, 36, 38] compared to a few prospective medium- and long-term studies, usually with a low number of patients. One prospective split-mouth study assessed the quality and the cumulative survival rates of CGPC (93.3%) compared with CIO (88.8%) from 29 patients after 5.5 years [31]. Another split-mouth study revealed a survival rate of 97% or more in posterior teeth restored with two types of ceramic onlays from 25 patients at the 7-years follow-up [50]. There are two prospective clinical studies assessing the success rates of ceramic in- and onlays with a maximum of 96 restorations after 12 years [26, 32]. Further, there is one prospective study revealing a success rate of 75.9% after a 15-years follow-up in 252 partial and complete all-ceramic coverage restorations from 121 patients [28]. In a prospective non-randomized clinical study all 103 occlusal lithium disilicate onlays were in function at the 11-years follow-up from seven patients suffering severe tooth wear [51].

In a review, medium-term survival rates were evaluated for ceramic onlays with 91 to 100% after two up to five years [52]. The long-term survival rates decreased to 71—98.5% after more than five years. Interestingly, neither the fabrication materials, the methods, nor the adhesive bonding systems seemed to affect longevity. The ceramic thickness of at least 2 mm and a retentive preparation design were evaluated to be more crucial. Further, ceramic failures were more often in non-vital teeth, posterior teeth and teeth from patients with parafunctional habits. A recently published meta-analysis revealed a pooled overall survival for ceramic onlays with 89.2% after five years including four studies [49]. Malament et al. [53] revealed a remarkable high estimated cumulative survival rate of e.max lithium disilicate glass ceramic onlays with 98.3% at 9.8 years. These authors showed additional data with an estimated cumulative survival rate of 95% in posterior complete and partial coverage ceramic restorations at 16.9 years [54]. Interestingly, the 10-years survival rate of CGPCs (*n* = 1679) was reported with 86.1% compared to estimated rates of other studies with a range of 70—96% [23]. Largely consistent with current literature, in the present study the survival estimates of CIOs and CGPCs from 325 patients were similar with 93.9 after 15.2 years and 92.6% after 14.9 years. However, the difference of the higher success rates of CIO (92.1%) compared to the lower rates of CGPC (84.2%) after 14.5 years was minimized obviously with follow-up times of more than 20 years.

Metal restorations, such as CGPC, do have a wide range of clinical applications since decades. They are characterized by a well-established manufacturing process in dental laboratory and must be considered as clinically proven. Modern dentistry does focus more and more on the preservation of dental hard tissue, a high biocompatibility of dental materials and the patient’s wish for tooth colored restorations in order to obtain good esthetic results. Teeth restored with ceramic partial coverage restorations are able to meet these requirements. However, the depth of the tooth cavity is still one crucial parameter when selecting the most suitable type of restoration, particular in case of defects below the cementoenamel junction (CEJ). The more subgingival the margin of the sound dental hard tissue is located, the more challenging is the reliable use of an adhesive technique, which is needed to insert a CIO properly. In our department operators rather preferred to restore teeth with subgingival cavities with CGPC than CIO, especially in the upper molars. To overcome deep-cavity associated restrictions of restoring posterior teeth with ceramics, deep margin elevation was introduced, e.g. in case of ceramic and resin composite inlays [55, 56]. Hereby the operator builds up the deepest dentinal cavity with a few layers of composite in order to allow placing the indirect restoration’s margin superior to the CEJ. A recent study demonstrated an overall cumulative survival rate of 95% with a mean observation time of 4.8 years in such restored teeth [57]. Besides the higher rate of biofilm accumulation on composite surfaces compared to ceramic surfaces, significantly more degradation of the composite build-ups was shown over time. Thus, one might suppose there is a high risk for the formation of secondary caries as a potential complication in the long-term. However, until now, the current evidence, mainly based on laboratory studies and limited clinical data, indicates that the deep marginal elevation can be a promising approach to restore teeth with localized subgingival defects resulting in good periodontal health [58]. In the present study, this specific concept was not implemented. According to the former university’s teaching concept in restorative dentistry, molars with extremely deep localized subgingival defects were restored more likely with CGPCs. The data highlight the outstanding long-term survival of teeth restored with both types, CIO or CGPC.

**Fig.3:**
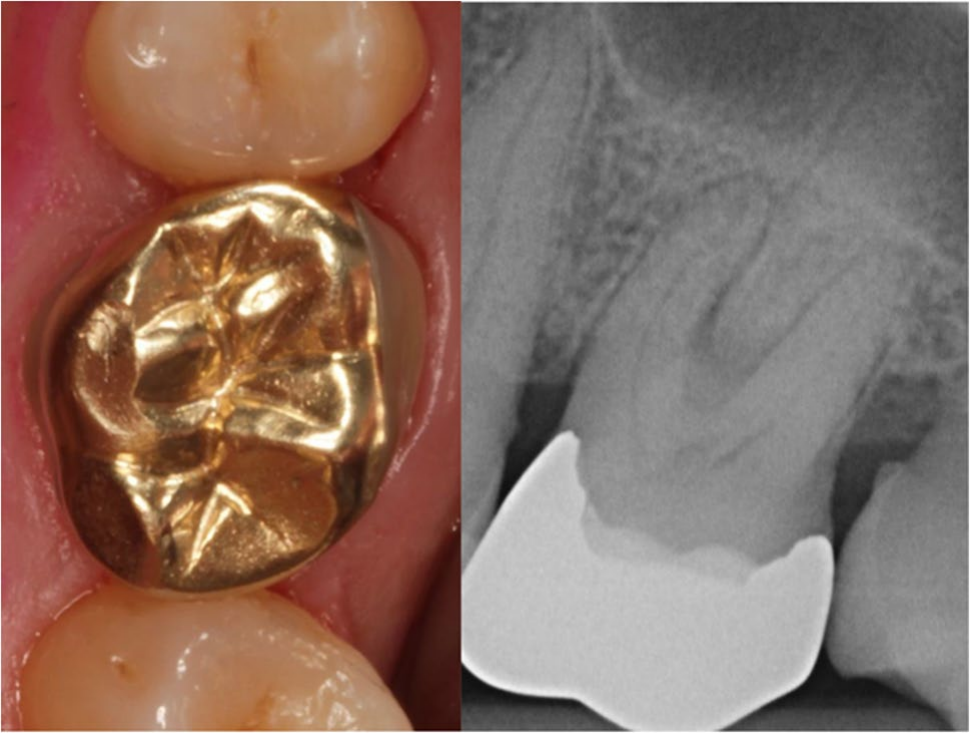
19.6-year follow-up of tooth 26 restored with cast gold partial crown showing good quality (sum of scores: 18) and loss of marginal bone

Interestingly, in cases of restoring posterior teeth with CIO, it was reported that failures do occur commonly in the molar region [53]. In the present study, the proportion of molars in teeth restored with CGPC or CIO was 89.6 or 71.4%. Taking into account the above mentioned more challenging restorative condition in molars restored with CGPC and the higher number of such teeth compared to the molars restored with CIO, both cumulative success estimates are close to each other with 82.9% (CGPC) and 89.3% (CIO) after 23.8 and 22.4 years. Fig. 3

**Fig.4:**
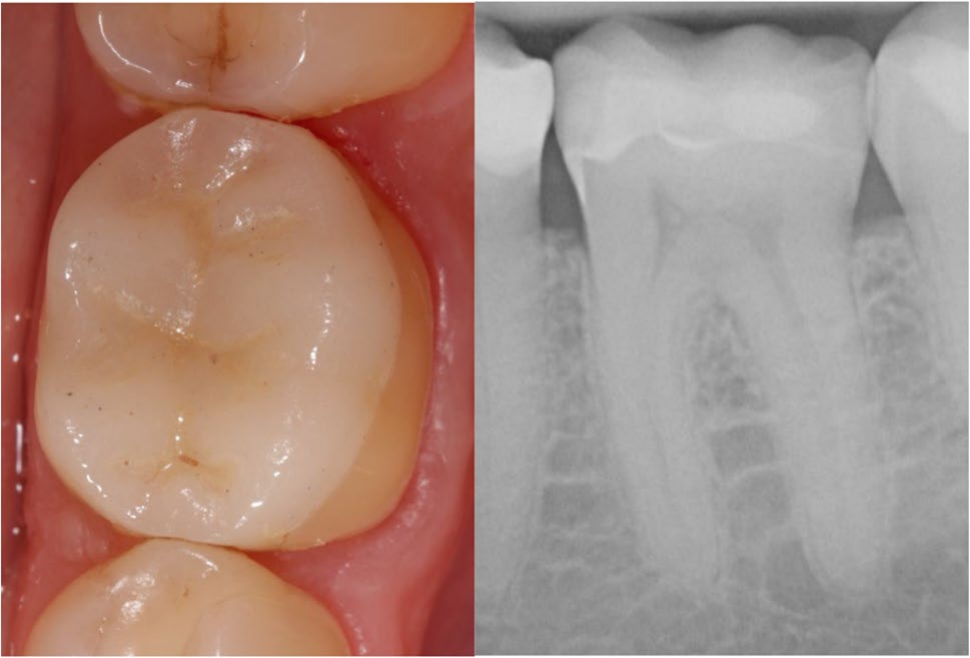
17.6-year follow-up of tooth 36 restored with ceramic partial crown showing good quality (sum of scores: 18) and sound marginal bone

In the present study, secondary caries was the significant predictor for a probability model predicting failure with an odds ratio of 6.6. The model included restorative and endodontic complications, secondary caries, patient’s age, age of restoration, and gender. Endodontic complications occurred three times more often in CGPCs than in CIOs. Survival of restorations was not associated with endodontic complication, rather with secondary caries and ceramic fracture. However, the root canal treated tooth is deemed to be a substantial risk factor for the survival of teeth with cast gold restorations [24]. In the present work, the performance of endodontic therapy affected significantly the estimate of success, however, pre- and postoperatively, there was a low rate of failures. Adverse events e.g. chipping or fracture of the ceramic restoration is common and well-reported, mainly occurring as an early complication [59]. In the long-term operator- and patient-related factors have a higher impact on treatment outcome than restorative therapy choices. Periodontal and endodontic reasons, which may lead to an early tooth loss, must be evaluated carefully. Within the limitations of this retrospective study, it is possible to overestimate the promising long-term success rates of both restoration types from the recalled patients due to a high rate of censored restorations within the observation period. However, a restorative concept of high quality and the need for professional tooth cleaning supporting the patient’s skills maintaining good oral health seem to be crucial in order to obtain high success rates of dental restorations in the long-term. Fig. 4

## Conclusions

CIOs and CGPCs achieved high cumulative success rates with 92.1 and 84.2% after 14.5 years performed by supervised undergraduate students. After up to 24.8 years, there were excellent and good qualities with a rate of 68% in teeth restored with CGPC and 71.8% in those restored with CIO. The longevity of the restorations may benefit from the intraoral repair of accessible defects and, in case of pulp infection or necrosis, an adequate endodontic management.

Clinical relevance: CIOs and CGPCs made by supervised undergraduate students are proper restoration types in posterior teeth in the long-term. An adequate preparation design, meticulous care in the inserting technique and constant biofilm removal due to proper oral hygiene combined with professional maintenance care are substantial. The clinical long-term performance was mostly limited by ceramic fractures in CIOs and endodontic complications in CGPCs.
